# Naevi: Report of a Case and Treatment by Electrolysis*Read at meeting of East Texas Medico-Chirurgical Society, November, 1901.

**Published:** 1902-03

**Authors:** R. M. Dunn

**Affiliations:** Palestine, Texas


For Texas Medical Journal.

### Naevi: Report of a Case and Treatment by Electrolysis.*

BY R. M. DUNN, M. D., PALESTINE, TEXAS.

It is not my intention to include in this short paper all forms of naevi; neither shall I go deeply into their etiology or pathology, because neither has much influence on the mode of treatment to be presented. It is generally supposed by most writers on the subject that all naevi are caused from some nervous manifestation of the mother during intrauterine life.

I however, saw a well marked nevus the size of the end of the little finger, on the right lip of the meatus urinaris of a respectable young farmer of this county. I do not know how that could have been a mother's mark.

As to their pathology, naevi differ greatly, according to their cause and situation. Those of the true skin are mostly pigmentary, while those of the mucous and muco-cutaneous surfaces are mostly erectile, and composed of dilatations of the capillaries with an ex-

*Read at meeting of East Texas Medico-Chirurgical Society, November, 1901.

pansile connective tissue strama. In some the veins predominate, while in others the arteries are more numerous and dilatable with their walls.

The treatment of this affection by electrolysis was in vogue more than a half century ago, but owing to poor instruments no correct way by which to measure the current, and an inadequate knowledge of the effect of electricity, the subject never received much attention till within the past fifteen or twenty years.

Case, Mattie McK., age 6 years, white. Shortly after birth a small, rather red elevation was noticed on the middle of the mucous surface of the upper lip, which increased in size when the child cried, and gradually grew larger as the child advanced in age. When I saw her, on November 8, 1998, the tumor was the size of a large pecan, and extended equally on either side of the median line. On November 9, 1898, Dr. Jamison administered chloroform, and I inserted at the base of the tumor four needles from each pole of a No. 2 McIntosh table plate with a galvanic current. The needles were inserted in opposite directions; that is, those from the negative on one side, while those from the positive were on the other: Twelve milliamperes were then gradually turned on and kept flowing for ten minutes, the poll changer being reversed twice. The tumor looked blanched, and felt hard.

This was repeated at intervals of three days until four treatments had been given; the current, however, had been increased to fifteen milliamperes and the time to eighteen minutes. She was then sent home, with instructions to return in six weeks.

We next treated her on December 27, this time using on the negative electrode a circular disc containing eighteen fine, short steel needles. The positive pole, a large, flat sponge, was placed on the thigh. The current was allowed to flow for fifteen minutes, and the quantity was eighteen milliamperes. The disk was inserted on one edge of the tumor, and when the current had been turned off, the needle withdrawn and reinserted at a different place, preferably on the opposite side or end, and the same treatment carried out as at first. Owing to sickness in the family, her mother was compelled to take her home, and she had only two treatments (four insertions of the disc).

When she returned, on February 20, 1899, the tumor was perceptibly smaller. She had four treatments, the same as those in December.

I have not seen her since, but letters from her parents indicate that the tumor is entirely gone; besides, Dr. Jamison saw her more than a year after the last treatment, and he says she is entirely relieved.

This case represents closely what you will have to do in most cases of the erectile type; however, you will find much difference in the reaction and resisting power of individual tissue and persons. I have a case under treatment at the present time, situated in exactly the same place, and a little larger, the girl being fourteen years of age, that only requires ten milliamperes for ten minutes. The flat pigmentary and port wine variety will require less current and shorter time. Strict asepsis is essential to success.

